# A new sight to acute pancreatitis through paracolic gutter exudation, a multicenter retrospective study

**DOI:** 10.1016/j.heliyon.2024.e29531

**Published:** 2024-04-10

**Authors:** Lianjie Lin, Tao Liu, Bingli Deng, Hongzong Fu, Xuelian Xiang, Zhihai Liang, Dongsheng Liang, Guodu Tang

**Affiliations:** a Department of Gastroenterology, The First Affiliated Hospital of Guangxi Medical University, Nanning, PR China; b Department of Spleen, Stomach and Hepatology, Guangxi International Zhuang Medicine Hospital, Nanning, PR China; c Department of Gastroenterology, The Second People's Hospital of Qinzhou, Guangxi, PR China

**Keywords:** Acute pancreatitis, Paracolic gutter exudation, Severity, Prognosis, CT

## Abstract

**Objectives:**

Paracolic gutter exudation (PGE) may influence the severity of acute pancreatitis, but no study has explored it extensively. The objective of this study was to evaluate PGE for assessing the severity of disease.

**Methods:**

We performed a retrospective analysis of 488 patients from three tertiary hospitals in Guangxi, China. General clinical information, severity, and clinical courses were recorded. The PGE score were classified as follows: 0 for no exudation, 1 for unilateral exudation, and 2 for bilateral exudation. We used ROC curves to assess the predictive value of the PGE score, and logistic regression analysis to determine risk factors associated with death, ICU admission, and the occurrence of MODS.

**Results:**

This study included 352 patients with moderately severe acute pancreatitis (MSAP) and 136 patients with severe acute pancreatitis (SAP). Patients who had PGE experienced higher total hospitalization costs, longer hospital stays, a higher incidence of SAP, higher mortality rates, higher ICU admission rates, a higher incidence of MODS, and higher incidence of infections than those without (*P* < 0.05). Diagnostic efficacy in predicting severity in patients with MSAP and SAP increased after BISAP, MCTSI, modified Marshall, and SOFA scores combined with PGE score respectively. The PGE score of >1 is an independent risk factor for ICU admission and MODS occurrence. (*P* < 0.05).

**Conclusion:**

The PGE provides reliable and objective information for assessing severity and clinical course of patients with MSAP and SAP.

## 1 Introduction

Acute pancreatitis (AP) is the most common pancreatic disease, occurring at an annual incidence rate of 34.8 per 100,000 individuals [1]. Approximately 12 % of AP cases are categorized as severe acute pancreatitis (SAP), with a mortality rate reaching up to 35 %, imposing a significant economic burden on both the nation and its citizens [2,3]. Therefore, accurately classifying AP patients based on the severity of disease and timely identifying and managing clinical factors that may contribute to the progression of AP to SAP can improve the prognoses of AP patients.

Various clinical and imaging scores are widely employed to assess the severity of AP. Presently, the Modified CT Severity Index (MCTSI) score [4] stands as the most comprehensive imaging score. However, the score for evaluating pancreatic inflammatory response fails to adequately consider the impact of exudation location and extent on disease severity. Moreover, the clinical scores, such as severity in acute pancreatitis (BISAP) score [5], modified Marshall score [6] and sequential organ failure assessment (SOFA) score [7], mainly focus on assessing organ function and lack imaging evidences. Thus, they fail to comprehensively reflect pathological manifestations such as pancreatic inflammatory response and pancreatic necrosis. In recent years, new imaging features have been suggested as predictors of the severity of AP. Acute fluid collections (AFCs), which are localized complications of AP, areobserved in 30 %–50 % of patients [8]. Heiss et al. [9] demonstrated a significant correlation between the presence of distal fluid collections (pararenal posterior hiatus or paracolic gutter) and mortality, indicating that paracolic gutter exudation (PGE) may play a potentially significant role in evaluating the severity of AP. However, upon separate analysis of the left and right sides, no significant correlation with mortality was observed, leading to an uncertain conclusion. The relationship between PGE and the severity of AP has not been extensively investigated. Thus, this study retrospectively analyzes the clinical data of patients with moderately severe acute pancreatitis (MSAP) and SAP across three medical centers to assess the potential significance of PGE in evaluating the severity of disease in these patients.

## 2 Methods

### 2.1 Study population

This study retrospectively investigated a cohort of patients who were admitted to three hospitals in Guangxi, China: the First Affiliated Hospital of Guangxi Medical University from June 2017 to May 2022, the Guangxi International Zhuang Medicine Hospital from July 2019 to April 2023, and the Second People's Hospital of Qinzhou from January 2021 to May 2022. The previous study, conducted by our team, revealed that the number of acute peripancreatic fluid collection areas increased within one week of onset, and subsequently reduced [10]. Based on the finding, the inclusion criteria for our study involved patients who were admitted to the hospital within one week from the onset of AP and had undergone computed tomography (CT) scans. Disease severity was classified using the 2012 revision of the Atlanta Classification for AP [6]. The exclusion criteria were established as follows: mild acute pancreatitis (MAP), minors, pregnancy, cancer, immune disorders, severe organ impairment prior to disease onset, traumatic pancreatitis, pancreatitis after transendoscopic retrograde cholangiopancreatography, history of pancreatic surgery, and outpatient therapeutic measures that affect the determination of exudation extent. The study was approved by the Institutional Ethics Committee.

### 2.2 CT scan protocol

CT scans was performed using either a Siemens spiral CT machine from Germany or a GE spiral CT machine from the United States, covering the region from the diaphragm to the lower abdomen. All CT scans were reviewed by two senior radiologists. In cases where a consensus cannot be reached, the final decision was made after further discussion. Patients were classified into two groups based on the presence or absence of PGE, as assessed by CT scan. The group with PGE was subsequently sub-divided into two groups: those with unilateral and bilateral PGE. The PGE score is based on the degree of exudation as follows: 0 for no exudation, 1 for unilateral exudation, and 2 for bilateral exudation. It was scored at the maximum amount within one week from the onset of AP.

### 2.3 Data collection

Baseline and clinical characteristics were collected, including age, gender, history of smoking and drinking, basic medical history, etiology, hospitalization days, hospitalization cost, bedside index for BISAP score within 24 h of admission, modified Marshall score within 24 h of admission, SOFA score within 24 h of admission, the initial MCTSI score, and severity classification of AP. Clinical courses included death, admission to the intensive care unit (ICU), occurrence of multiple-organ dysfunction syndrome (MODS) [11], occurrence of infection (including pancreatic, peripancreatic, and extrapancreatic infections), usage of antimicrobials with special use-grade, implementation of percutaneous catheter drainage (PCD) [12], and utilization of surgical intervention.

### 2.4 Definitions

In terms of etiologic classification, the term “other or unknown” refers to causes that are not related to biliary, hypertriglyceridemic, alcoholic, or the causes cannot be determined according to clinical information. The term “deaths” included in-hospital deaths due to AP severity or deaths after abandonment of treatment due to AP severity. If any of the following criteria are met, a patient with AP is considered to have an infection: a). presence of symptoms and signs indicating infection, including systemic symptoms and signs (such as chills, shivering, fever, etc.) and local symptoms and signs (for example, lung manifestations of cough, sputum production, moist rales auscultated, etc.; abdominal manifestations of abdominal pain, tenderness, rebound tenderness, absent bowel sounds, etc., and/or other organ manifestations); b). positive culture of pathogenic microorganisms; c). imaging results indicative of infection, such as the presence of a bubble sign in the region of the pancreas and inflammatory exudation in the lungs; d). elevated levels of infection-related markers, including white blood cells, neutrophil percentage, C-reactive protein, and procalcitoninogen. The treatment of PCD refers to the removal of AFCs by selecting an appropriate location to enter the abdominal or pelvic cavity and placing a drainage tube under ultrasound or CT guidance. Surgical intervention refers to the removal of necrotic pancreatic tissues via laparoscopy or open abdomen surgery.

### 2.5 Statistical analysis

Statistical analysis was performed using the statistical software SPSS (version 26.0). Continuous variables with normal distribution were expressed as means ± standard deviations, and the differences between two groups were analyzed using Student's t-test. Continuous variables with non-normal distribution were expressed as medians with interquartile range (IQR), and Mann–Whitney U tests were used. Categorical variables were described as frequencies with percentages and compared using chi-square test or Fisher's exact test. The predictive value of the PGE score in patients with MSAP and SAP was assessed using ROC curves. Potential risk factors for death, ICU admission, and occurrence of MODS in these patients were determined by logistic regression analysis. A *P* value of <0.05 was considered statistically significant.

## 3 Results

A total of 352 patients with MSAP and 136 patients with SAP were included in this study. The baseline characteristics of these patients are presented in Table 1. The male-to-female ratio was approximately 2.39:1, with 344 males (70.5 %) and 144 females (29.5 %). The age ranged from 20 to 88 years old, and the median age was 45 years old (IQR 36–58 years old). The proportion of females was higher in the group with PGE than without (34.9 % vs. 22.8 %; *P* < 0.05). However, when comparing the groups with unilateral and bilateral exudation, the difference in the proportion of females was not statistically significant (*P* > 0.05).

**Table 1:** Baseline characteristics of 352 patients with MSAP and 136 patients with SAP.

Parameters	Total (n = 488)	PGE		unilateral or bilateral PGE		^*1*^*P* value	^*2*^*P* value
		Absent (n = 219)	Present (n = 269)	Unilateral (n = 121)	Bilateral (n = 148)		
Gender						＜0.05	＞0.05
Male	344(70.5 %)	169(77.2 %)	175(65.1 %)	74(61.2 %)	101(68.2 %)		
Female	144(29.5 %)	50(22.8 %)	94(34.9 %)	47(38.8 %)	47(31.8 %)		
Median age (Years, IQR)	45(36–58)	45(35–58)	45(36–57)	45(35–60)	45(36–56)	＞0.05	＞0.05
Median hospitalization costs (RMB, IQR)	19,803 (11,332–43,837)	14,715 (9330–24,472)	27,379 (14,455–55,376)	17,223 (9834–35,332)	44,895 (20,916–80,247)	＜0.05	＜0.05
Median hospitalization days (Days, IQR)	10(7–15)	8(6–12)	12(8–17)	10(7–14)	14(9–22.5)	＜0.05	＜0.05
Smoking history	184(47.7 %)	99(45.2 %)	116(43.1 %)	55(45.5 %)	61(41.2 %)	＞0.05	＞0.05
Drinking history	225(46.1 %)	99(45.2 %)	126(46.8 %)	61(50.4 %)	53(43.9 %)	＞0.05	＞0.05
Hypertension	123(25.2 %)	57(26.0 %)	66(24.5 %)	26(21.5 %)	40(27.0 %)	＞0.05	＞0.05
Diabetes	111(22.7 %)	54(24.7 %)	57(21.2 %)	24(19.8 %)	33(22.3 %)	＞0.05	＞0.05
Fatty liver	180(36.9 %)	76(34.7 %)	104(38.7 %)	46(38.0 %)	58(39.2 %)	＞0.05	＞0.05
Hyperlipidemia	272(55.7 %)	123(56.2 %)	149(55.4 %)	69(57.9 %)	80(54.1 %)	＞0.05	＞0.05
Etiology						＞0.05	＞0.05
Gallstones	155(31.8 %)	75(34.2 %)	80(29.7 %)	35(28.9 %)	45(30.4 %)		
Hypertriglyceridemia	161(33.0 %)	73(33.3 %)	88(32.7 %)	40(33.1 %)	48(32.4 %)		
Alcoholic	52(10.7 %)	18(8.2 %)	34(12.6 %)	13(10.7 %)	21(14.2 %)		
Mixed	35(7.2 %)	15(6.8 %)	20(7.4 %)	9(7.4 %)	11(7.4 %)		
Other or unclear	85(17.4 %)	38(17.4 %)	47(17.5 %)	24(19.8 %)	23(15.5 %)		

^1^*P* value, Absent vs. Present;^2^*P* value, unilateral vs. bilateral.

The median hospitalization cost was RMB 19,803 (IQR RMB 11,332 to RMB 43,837), and the median number of hospitalization days was 10 days (IQR 7–15 days). Patients in the group with PGE had higher hospitalization costs (RMB 27,379 vs. RMB 14,715; *P* < 0.05) and longer hospitalization days than without (12 days vs. 8 days; *P* < 0.05). The difference was statistically significant in the comparison of the unilateral and bilateral groups (RMB 17,223 vs. RMB 44,895; 10 days vs. 14 days; *P* < 0.05).

### 3.1 Relationship between PGE score and BISAP, MCTSI, modified marshall, and SOFA scores

The MCTSI scores in patients with MSAP and SAP increased with the PGE score (*P* < 0.05; Table 2). Moreover, patients with a PGE score of 2 had higher BISAP, modified Marshall, and SOFA scores compared to those with a score of 1 (*P* < 0.05; Table 2).

**Table 2:** Comparison of the common scores of MSAP and SAP patients with different PGE scores.

Parameters	0 points (n = 219)	1 point (n = 121)	2 points (n = 148)	*P* value	^*1*^*P* value	^*2*^*P* value	^*3*^*P* value
BISAP score	1(0–2)	1(0–2)	2(1–3)	＜0.05	＞0.05	＜0.05	＜0.05
MCTSI score	4(2–6)	6(6–8)	8(6–8)	＜0.05	＜0.05	＜0.05	＜0.05
Modified Marshall score	0(0–2)	1(0–2)	2(0–2)	＜0.05	＞0.05	＜0.05	＜0.05
SOFA score	1(0–3)	1(0–3)	3(2–4)	＜0.05	＞0.05	＜0.05	＜0.05

*P* value, comparison among the three groups; ^*1*^*P* value, 0 points vs. 1 point; ^*2*^*P* value, 0 points vs. 2 points; ^*3*^*P* value, 1 point vs. 2 points.

### 3.2 Disease severity and clinical course in patients with and without PGE

Within a cohort of 488 patients, it was observed that 55.1 % of the individuals exhibited PGE. Patients with PGE showed a higher incidence of SAP (40.1 % vs. 12.8 %; *P* < 0.05), higher mortality rates (8.9 % vs. 1.4 %; *P* < 0.05), higher ICU admission rates (27.1 % vs. 6.8 %; *P* < 0.05), a higher incidence of MODS (21.6 % vs. 6.4 %; *P* < 0.05), a higher incidence of infections (90.7 % vs. 79.0 %; *P* < 0.05), higher rate of usage of antimicrobials with special use-grade (33.5 % vs. 13.2 %, *P* < 0.05), higher proportion of implementation of PCD (15.6 % vs. 1.4 %; *P* < 0.05), and higher proportion of utilization of surgical intervention (3.0 % vs. 0.5 %, *P* < 0.05) than the patients without(Table 3).

**Table 3:** Comparison of the disease severity and clinical course of MSAP and SAP patients with different PGE degree.

Parameters	Total (n = 488)	PGE		unilateral or bilateral PGE		^*1*^*P* value	^*2*^*P* value
		Absent (n = 219)	Present (n = 269)	Unilateral (n = 121)	Bilateral (n = 148)		
SAP	136(27.9 %)	28(12.8 %)	108(40.1 %)	28(23.1 %)	80(54.1 %)	＜0.05	＜0.05
Death	27(5.5 %)	3(1.4 %)	24(8.9 %)	5(4.1 %)	19(12.8 %)	＜0.05	＜0.05
ICU admission	88(18.0 %)	15(6.8 %)	73(27.1 %)	14(11.6 %)	59(39.9 %)	＜0.05	＜0.05
MODS occurrence	72(14.8 %)	14(6.4 %)	58(21.6 %)	12(9.9 %)	46(31.1 %)	＜0.05	＜0.05
Infection	417(85.5 %)	173(79.0 %)	244(90.7 %)	104(86.0 %)	140(94.6 %)	＜0.05	＜0.05
Usage of antimicrobials with special use-grade	119(24.4 %)	29(13.2 %)	90(33.5 %)	17(14.0 %)	73(49.3 %)	＜0.05	＜0.05
Implementation of PCD	45(9.2 %)	3(1.4 %)	42(15.6 %)	5(4.1 %)	37(25.0 %)	＜0.05	＜0.05
Utilization of surgical intervention	9(1.8 %)	1(0.5 %)	8(3.0 %)	1(0.8 %)	7(4.7 %)	＜0.05	＞0.05

^1^*P* value, Absent vs. Present; ^2^*P* value, unilateral vs. bilateral.

### 3.3 Disease severity and clinical course in patients with unilateral and bilateral PGE

Among the 269 patients with PGE, 45.0 % exhibited unilateral PGE, and 55.0 % exhibited bilateral PGE. Patients with bilateral PGE showed a higher incidence of SAP (54.1 % vs. 23.1 %; *P* < 0.05), higher mortality rates (12.8 % vs. 4.1 %; *P* < 0.05), higher ICU admission rates (39.9 % vs. 11.6 %; *P* < 0.05), a higher incidence of MODS (31.1 % vs. 9.9 %; *P* < 0.05), a higher incidence of infections (94.6 % vs. 86.0 %; *P* < 0.05), higher rate of usage of antimicrobials with special use-grade (49.3 % vs. 14.0 %, *P* < 0.05), and a higher proportion of utilization of surgical intervention (25.0 % vs. 4.1 %; *P* < 0.05) than the patients with unilateral (Table 3).

### 3.4 PGE, BISAP, MCTSI, modified marshall, and SOFA scores in predicting the severity of MSAP and SAP

**Fig. 1:**
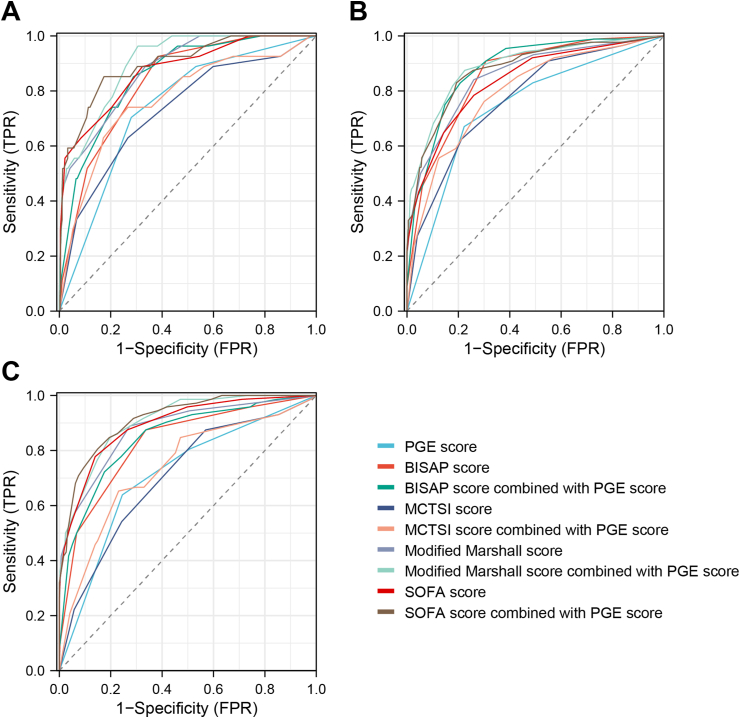
ROC curve of different scoring system in predicting poor prognosis of MSAP and SAP patients. (A) death; (B) ICU admission; (C) MODS occurrence.

In terms of death, the sequence of area under the curve (AUC) values for each score in descending order was as follows: modified Marshall score > SOFA score > BISAP score > PGE score > MCTSI score. The AUC values were 0.876 (95 % CI: 0.817–0.935), 0.873 (95 % CI: 0.801–0.945), 0.832 (95 % CI: 0.767–0.897), 0.741 (95 % CI: 0.653–0.830), and 0.732 (95 % CI: 0.629–0.835) respectively, and the diagnostic cutoffs were >1, >2, >1, >1, and >6, respectively. The diagnostic efficacy of the MCTSI and modified Marshall scores were improved after combining with PGE score, and the AUC values were 0.772 (95 % CI: 0.673–0.871) and 0.902 (95 % CI: 0.856–0.948) after the combination, respectively. The difference was statistically significant (*P* < 0.05; Table 4; Fig. 1A).

**Table 4:** ROC curve results of PGE, BISAP, MCTSI, modified Marshall, and SOFA scores in predicting death, ICU admission and MODS occurrence in patients with MSAP and SAP.

Parameters	AUC	95%CI	Diagnosis cutoff	Sensitivity	Specificity	^*1*^*P* value	^*2*^*P* value
Death							
PGE score	0.741	0.653–0.830	>1	70.4	72.0	＜0.05	–
BISAP score	0.832	0.767–0.897	>1	92.6	61.4	＜0.05	＞0.05
BISAP score combined with PGE score	0.854	0.791–0.918	–	85.2	71.1	＜0.05	
MCTSI score	0.732	0.629–0.835	>6	63.0	73.3	＜0.05	＜0.05
MCTSI score combined with PGE score	0.772	0.673–0.871	–	74.1	73.3	＜0.05	
Modified Marshall score	0.876	0.817–0.935	>1	88.9	66.6	＜0.05	＜0.05
Modified Marshall score combined with PGE score	0.902	0.856–0.948	–	96.3	69.4	＜0.05	
SOFA score	0.873	0.801–0.945	>2	88.9	67.7	＜0.05	＞0.05
SOFA score combined with PGE score	0.898	0.835–0.960	–	85.2	82.6	＜0.05	
ICU admission							
PGE score	0.742	0.684–0.800	>1	67.1	77.7	＜0.05	–
BISAP score	0.855	0.814–0.895	>1	90.9	69.2	＜0.05	＜0.05
BISAP score combined with PGE score	0.882	0.845–0.919	–	83.0	79.2	＜0.05	
MCTSI score	0.771	0.717–0.825	>6	62.5	78.7	＜0.05	＜0.05
MCTSI score combined with PGE score	0.795	0.743–0.847	–	76.1	70.0	＜0.05	
Modified Marshall score	0.855	0.809–0.901	>1	84.1	74.0	＜0.05	＜0.05
Modified Marshall score combined with PGE score	0.889	0.849–0.929	–	87.5	77.5	＜0.05	
SOFA score	0.838	0.790–0.887	>2	78.4	74.0	＜0.05	＜0.05
SOFA score combined with PGE score	0.878	0.836–0.92	–	83.0	80.8	＜0.05	
MODS occurrence							
PGE score	0.712	0.647–0.778	>1	63.9	75.5	＜0.05	–
BISAP score	0.829	0.776–0.882	>1	87.5	66.3	＜0.05	＜0.05
BISAP score combined with PGE score	0.848	0.797–0.898	–	72.2	82.5	＜0.05	
MCTSI score	0.706	0.641–0.771	>4	87.5	43.0	＜0.05	＜0.05
MCTSI score combined with PGE score	0.739	0.674–0.805	–	65.3	76.9	＜0.05	
Modified Marshall score	0.878	0.831–0.924	>1	88.9	72.6	＜0.05	＜0.05
Modified Marshall score combined with PGE score	0.906	0.871–0.940	–	84.7	80.3	＜0.05	
SOFA score	0.893	0.852–0.934	>3	77.8	86.1	＜0.05	＜0.05
SOFA score combined with PGE score	0.914	0.880–0.947	–	80.6	85.1	＜0.05	

^1^*P* value, single scoring system.

^2^*P* value, AUC value after a scoring system combined with paracolic sulci exudate score was compared with that before no combination.

In terms of ICU admissions, the sequence of the AUC values of each score in descending was as follows: BISAP score = modified Marshall score > SOFA score > MCTSI score > PGE score. The AUC values were 0.855 (95 % CI: 0.814–0.895), 0.855 (95 % CI: 0.809–0.901), and 0.838 (95 % CI: 0.790–0.887), 0.771 (95 % CI: 0.717–0.825), and 0.742 (95 % CI: 0.684–0.800) respectively, and the diagnostic cutoffs were >1, >1, >2, >6, and >1, respectively. The diagnostic efficacy of BISAP, MCTSI, modified Marshall, and SOFA scores were improved after combining with PGE score. The highest diagnostic efficacy was achieved by the modified Marshall score combined with the PGE score, having an AUC value of 0.889 (95 % CI: 0.849 to 0.929). The difference was statistically significant (*P* < 0.05; Table 4; Fig. 1B).

In terms of MODS occurrence, the sequence of the AUC values of each score in descending order were as follows: SOFA score > modified Marshall score > BISAP score > PGE score > MCTSI score. The AUC values were 0.893 (95 % CI: 0.852–0.934), 0.878 (95 % CI: 0.831–0.924), and 0.829 (95 % CI: 0.776–0.882), 0.712 (95%CI: 0.647–0.778), and 0.706 (95%CI: 0.641–0.771) respectively. The diagnostic cutoffs were >3, >1, >1, >1, and >4, respectively. The diagnostic efficacy of BISAP score, MCTSI score, modified Marshall score, and SOFA score were improved after combining with PGE score, and the highest diagnostic efficacy was achieved by the SOFA score combined with the PGE score, with an AUC value of 0.914 (95 % CI: 0.880–0.947). The difference was statistically significant (*P* < 0.05; Table 4; Fig. 1C).

### 3.5 Potential risk factors of death, ICU admission, and MODS occurrence in patients with MSAP and SAP

The PGE, BISAP, MCTSI, modified Marshall, and SOFA scores were reassigned based on the diagnostic cutoff value. Scores higher than the cutoff value were assigned a value of 1, while scores lower than the cutoff value were assigned a value of 0. Binary logistic regression analyses were conducted to assess the risk factors of death, admission to ICU, and the occurrence of MODS in patients with MSAP and SAP. The results showed that BISAP score >1 and SOFA score >2 were independent risk factors for death in patients with MSAP and SAP. PGE score >1, BISAP score >1, MCTSI score >6, modified Marshall score >1, and SOFA >2 were established as independent risk factors for ICU admission in patients with MSAP and SAP. Additionally, PGE score >1, BISAP score >1, modified Marshall score >1 and SOFA score >3 were independent risk factors for MODS occurrence in MSAP and SAP patients (Table 5).

**Table 5:** Results of logistic regression analysis to determine potential risk factors of death, ICU admission, and MODS occurrence.

Parameters	B value	SE value	Wald value	OR value	95%CI	*P* value
Death						
PGE score	0.665	0.515	1.666	1.944	0.709–5.335	＞0.05
BISAP score	1.603	0.793	4.087	4.968	1.050–23.507	＜0.05
MCTSI score	0.496	0.494	1.011	1.642	0.624–4.321	＞0.05
Modified Marshall score	1.202	0.704	2.910	3.326	0.836–13.230	＞0.05
SOFA score	1.432	0.699	4.205	4.189	1.065–16.470	＜0.05
ICU admission						
PGE score	0.811	0.338	5.750	2.251	1.160–4.368	＜0.05
BISAP score	1.944	0.421	21.367	6.989	3.065–15.938	＜0.05
MCTSI score	0.934	0.339	7.579	2.545	1.309–4.950	＜0.05
Modified Marshall score	1.522	0.385	15.652	4.582	2.155–9.738	＜0.05
SOFA score	0.888	0.367	5.871	2.431	1.185–4.987	＜0.05
MODS occurrence						
PGE score	0.744	0.376	3.908	2.104	1.006–4.399	＜0.05
BISAP score	1.270	0.432	8.644	3.559	1.527–8.297	＜0.05
MCTSI score	0.517	0.487	1.129	1.677	0.646–4.352	＞0.05
Modified Marshall score	1.321	0.474	7.776	3.746	1.481–9.479	＜0.05
SOFA score	1.923	0.384	25.04	6.839	3.221–14.522	＜0.05

## 4 Discussion

AP is a common acute disease of the digestive system, and patients with mild acute pancreatitis can recover within one week. SAP patients are often accompanied by infection and MODS and even result in death, with mortality rate reaches up to 35 % [2,3]. Therefore, predicting the severity of AP is of great significance for individualized treatment. Currently, BISAP, modified Marshall, SOFA, MCTSI and other scores are widely used in clinical practice to predict the severity of AP. The application of CT can aid in more accurately assessing the extent and severity of lesions, as well as detecting involvement of extrapancreatic organs. However, these scoring systems have some shortcomings. For instance, the clinical score does not include imaging data that can accurately reflect pancreatic inflammation and necrosis. Meanwhile, MCTSI score is a more comprehensive imaging score, but it does not consider the impact of location and extent of exudation in evaluating pancreatic inflammatory response. In 2010, Heiss et al. [9] reported that the presence of fluid aggregation in the distal area (pararenal posterior interstitial space or paracolic gutter) on CT imaging had a significant correlation with mortality. However, the authors found no correlation with mortality when they analyzed left and right PGE separately, possibly due to the small number of subjects or the low level of imaging technology. Furthermore, their study did not investigate the relationship between unilateral and bilateral PGE and disease severity, which has certain limitations.

The BISAP, MCTSI, modified Marshall, and SOFA scores can be used to assess the severity of AP and predict the occurrence of adverse clinical outcomes [[13], [14], [15]]. This study demonstrates that there is a great consistency between the PGE score and the above scoring system, indicating that the AP patients with bilateral PGE experience a more severe illness state. These results are closely related to the pathophysiological mechanism. In the acute period of AP, released inflammatory factors, cell damage and increased vascular permeability combine to initiate capillary leak syndrome. This results in the infiltration of pancreatic enzymes, albumin, inflammatory factors, and other substances from the vasculature into the third interstitial space. As the disease progresses, factors such as the rupture of the pancreatic ducts, hypoproteinemia and portal hypertension may be involved in the formation of exudation, particularly in patients with SAP [16]. Due to the dynamic nature of the fluid and the histolytic effect of pancreatic enzymes, AFCs can disseminate through the anatomical space and even reach the paracolic gutter, leading to extensive exudation [17]. Extensive exudation leads to increased intra-abdominal pressure and may even result in abdominal compartment syndrome, which affected the function of the heart, kidneys, lungs, and other important organs through pathophysiological changes, including reduced blood flow in right heart regurgitation, reduced cardiac output, reduced arterial perfusion pressure of the peripheral organs, and transmission of pressure, among others [18]. The absorption of inflammatory mediators and harmful substances assimilated into the bloodstream via the peritoneum can lead to systemic inflammatory response syndrome and sepsis, further exacerbating damage. Regarding therapeutic measures, patients with PGE underwent a greater number of PCD treatments, suggesting that the utilization of PCD serve as an effective means of treating patients with extensive exudation. However, further multicenter and prospective studies are needed to confirm its effectiveness and explore the best time of the treatment.

In this study, the result of ROC curves indicated that the diagnostic efficacy of the MCTSI score in predicting death, admission to ICU, and occurrence of MODS was lower than others, consistent with the findings from previous research [19]. However, it was observed that the diagnostic efficacy of the MCTSI score improved when combined with the PGE score. This improvement may be attributed to the fact that the severity of AP is relate to the extent of exudation. As an illustration, when the pancreatic inflammatory response of a patient is rated as 4 in the MCTSI score, the extent of exudation is not necessarily extensive. However, the addition of the PGE score can facilitate the assessment of the extent of exudation to a notable degree, resulting in a more precise score and thereby enhancing diagnostic efficacy. PGE as a presenting feature in CT, of which the score can provide complementary information to the image scoring system, indicating that PGE especially its extent should be considered when imaging scoring system or clinical scoring system were used to evaluate the severity of AP. In addition, PGE can also be detected by ultrasound [20], but its value in PGE may be interfered by intestinal gas. In evaluating complications of pancreatitis, ultrasound has certain limitations [20]. However, it is interesting to note that ultrasound may play a crucial guiding role in PCD treatment.

However, there were some limitations in this study. The volume of PGE was not precisely quantitative measured, which is needed for methods to calculate precise indicators that would be more beneficial to evaluate the severity of AP. Furthermore, external validation is still needed to better verify the universality of the findings.

## 5 Conclusion

In conclusion, our multicenter retrospective study reveals that AP patients with PGE have a tendency to develop SAP, and experience a more severe course of disease and poorer prognosis compared to those without PGE. The new simple scoring system proposed in this study to assess the extent of PGE is an important complementary information when performing assessment used image and clinical scoring system. PGE is an independent predictive factor for identifying the AP patients at risk and predicting the prognoses of AP patients.

## Funding

This study was supported by 10.13039/501100001809National Natural Science Foundation of China (81970558).

## Data availability statement

Data will be made available on request.

## Ethical declaration

The study was approved by the Ethical Review Committee of First Affiliated Hospital of Guangxi Medical University(2023-E396-01).

## CRediT authorship contribution statement

**Lianjie Lin:** Writing – original draft, Resources, Methodology, Investigation, Data curation. **Tao Liu:** Writing – original draft, Formal analysis, Data curation. **Bingli Deng:** Investigation, Data curation. **Hongzong Fu:** Investigation, Data curation. **Xuelian Xiang:** Investigation, Data curation. **Zhihai Liang:** Writing – review & editing, Project administration, Conceptualization. **Dongsheng Liang:** Writing – review & editing, Conceptualization. **Guodu Tang:** Writing – review & editing, Funding acquisition, Conceptualization.

## Declaration of competing interest

The authors declare that they have no known competing financial interests or personal relationships that could have appeared to influence the work reported in this paper.
